# An Easily Customized Gesture Recognizer for Assisted Living Using Commodity Mobile Devices

**DOI:** 10.1155/2018/3180652

**Published:** 2018-07-19

**Authors:** Antigoni Mezari, Ilias Maglogiannis

**Affiliations:** Department of Digital Systems, University of Piraeus, Piraeus, Greece

## Abstract

Automatic gesture recognition is an important field in the area of human-computer interaction. Until recently, the main approach to gesture recognition was based mainly on real time video processing. The objective of this work is to propose the utilization of commodity smartwatches for such purpose. Smartwatches embed accelerometer sensors, and they are endowed with wireless communication capabilities (primarily Bluetooth), so as to connect with mobile phones on which gesture recognition algorithms may be executed. The algorithmic approach proposed in this paper accepts as the input readings from the smartwatch accelerometer sensors and processes them on the mobile phone. As a case study, the gesture recognition application was developed for Android devices and the Pebble smartwatch. This application allows the user to define the set of gestures and to train the system to recognize them. Three alternative methodologies were implemented and evaluated using a set of six 3-D natural gestures. All the reported results are quite satisfactory, while the method based on SAX (Symbolic Aggregate approXimation) was proven the most efficient.

## 1. Introduction

Gesture recognition refers to recognizing meaningful body motions involving movements of the fingers, hands, arms, head, face, or body performed with the intent to convey meaningful information or to interact with the environment [1]. This topic is considered extremely important in applications based on smart and efficient human computer interfaces. More specifically, gesture recognition applies to several computer applications, such as those involving young children interaction [2, 3], sign language recognition [4, 5], monitoring of physical activity or events involving disabled persons or the elderly [6, 7], the medical monitoring of the emotional state or level of stress [8], the navigation or manipulation in virtual environments [9], the communicating in a teleconference [10], the distance learning [11], and the monitoring of driver alertness/drowsiness status [12].

The implementation of computerized gesture recognition requires the use of various visual or sensor devices to track the motion. Recognition based on the visual channel is currently the most widespread method of recognizing gestures. The visual recording devices are usually installed at a fixed location and the gesture recognition is restricted in confined space. Wearable devices used for visual recognition include glasses camera [13] and wrist-worn device with infrared spectral camera (IR) [14].

Recognizing the motion of the fingers is a special topic in gesture recognition. It is used in sign language [4, 5] as well as in virtual reality and robotics. Sensor devices such as gloves with sensors [15, 16] and electromyogram sensors (EMG) [4] are also used to capture finger movements. A sensor ring is another wearable device that has been proposed for recognizing finger gestures [17, 18]. The creation of gesture vocabularies to manipulate devices is also an interesting topic. Park and Han [19] propose an analytical approach to the creation of multitouch control-gesture vocabularies applicable to mobile devices.

A smartwatch equipped with accelerometer can provide information about the movement of the hand and may be used for recognizing gestures. The main advantage of using a smartwatch is that it does not impose restrictions to the user. In this context, its use does not impose any restriction in space, and the user is not forced to use a special-purpose sensor device, which would probably cause him some kind of discomfort.

A number of prototype (proof-of-concept) gesture recognition applications based on smartwatches or wrist-worn devices may be found in the literature. For instance, Bernaerts et al. in [20] propose a smartwatch application to allow a person to physically and virtually lock and unlock doors, to acquire room information, and to send virtual knocks. Zhang et al. [21] use wearable accelerometer sensors attached to both wrists to detect eating and drinking activities. Shoaib et al. [22] use two mobile phones to recognize the user's activity. One of the phones is attached on the user wrist to simulate a smartwatch. The accelerometer and gyroscope measurements are utilized for the recognition of 13 activities. Garcia-Ceja et al. [23] use acceleration data from a wristwatch in order to identify long-term activities. The results may be used as an indicator of how independent a person is and as a source of information to healthcare intervention applications.

The aim of this work is to examine whether simple and natural gestures can be reliably recognized using commodity devices like a smartwatch and a smartphone and propose specific methodologies to improve performance and accuracy. The rest of the paper is structured as following: in Section 2, we provide background information on gesture recognition methods and we present the Pebble smartwatch used in this work. In Section 3, the proposed methodology is described for developing the gesture recognition system, while in Section 4 use cases and the system evaluation with corresponding experimental results are presented. Finally, Section 5 presents future plans and concludes the paper.

## 2. Related Work and Background Information

Several techniques for gesture recognition using motion sensors exist in the literature. Wobbrock et al. [24] developed $1 recognizer, a 2-D unistroke recognizer designed for rapid prototyping of gesture-based user interfaces. They proposed a 4-step processing of the recorded path of the gesture: (i) resample into a fixed number of points evenly spaced along the path, (ii) rotate the path so that the line from the centroid of the path to the first point of the path to be parallel to the *x*-axis, (iii) scale the path (nonuniformly) to a reference square, and (iv) move the path so that its centroid is at (0,0). To compare the gesture with the templates, the mean value of the Euclidean distance between the corresponding points is computed. The authors propose the use of an iterative method to fine-tune the rotation angle. The gesture is recognized as the template having the minimum distance. Protractor [25] is another recognizer quite similar to $1. The gesture is not scaled in this case, and the fine-tune rotation angle is calculated so that the cosine distance between the two gestures minimizes. Protractor uses the inverse cosine distance as the similarity score. Kratz and Rohs [26] proposed $3 recognizer, a modification of the $1 recognizer to manipulate 3-D data. Kratz and Rohs [27] improved $3 recognizer by computing the fine-tune rotation angle using a method similar to the one used by Protractor. They named the new recognizer Protractor3D. $N recognizer [28] is built upon the $1 and is intended for the recognition of multistroke 2-D gestures. $N-protractor [29] is a variation of $N that embeds the technique of Protractor. $P [30] belongs to the same recognizer family with $1 and $N. $P does not represent gestures as ordered series of points but as unordered point-clouds. Another gesture recognition method is uWave [31]. It uses the data of a three-axis accelerometer. The time series of the accelerometer data is compressed by an averaging window and the new values are nonlinearly quantized. uWave employs dynamic time warping (DTW) to match two time series and the Euclidean distance as the distance function. uWave adapts its templates to deal with the gesture variations through the time. Xie and Pan [32] aim to improve the accuracy of gesture recognition. They employ a low-pass filter to smooth the data and use dynamic-threshold truncation to remove data recorded before the gesture actually starts and after the gesture actually ends. To the produced time series, they append the amplitudes of its fast Fourier transform (the 21 first values).  Table 1 summarizes the most prominent gesture recognizers.

In this work, we propose the utilization of a commodity smartwatch as the motion sensor. Smartwatches are equipped with a microdisplay, integrated sensors, and network connectivity. The Pebble smartwatch features a 3-axis accelerometer that produces integer data measured in milli-Gs. It is calibrated to measure a maximum acceleration of ±4G. Accelerometer data can be received in batches, to save CPU time and battery life. The programmer can set the accelerometer sampling rate in one of the four valid values (10 Hz, 25 Hz, 50 Hz, and 100 Hz) and also the number of samples per batch. The communication with an Android or iOs device is implemented using Bluetooth 4.0 (Bluetooth low energy) protocol. The programmer can reduce the sniff interval, the period during which the Bluetooth module may not exchange (ACL) packets, if an app requires reduced latency when sending messages. An open software development kit (SDK) is available to programmers. The SDK, referred to as PebbleKit, is available for smartphones running iOs or Android and allows the two-way communication between the Pebble watch and the smartphone. Adding to the above characteristics that the Pebble watch is available at low cost, less than 100€, it is best suited for this research effort.

## 3. Gesture Recognition Methodology

### 3.1. Selected Gestures, Measurements, and Recognition Methods

**Figure 1:**
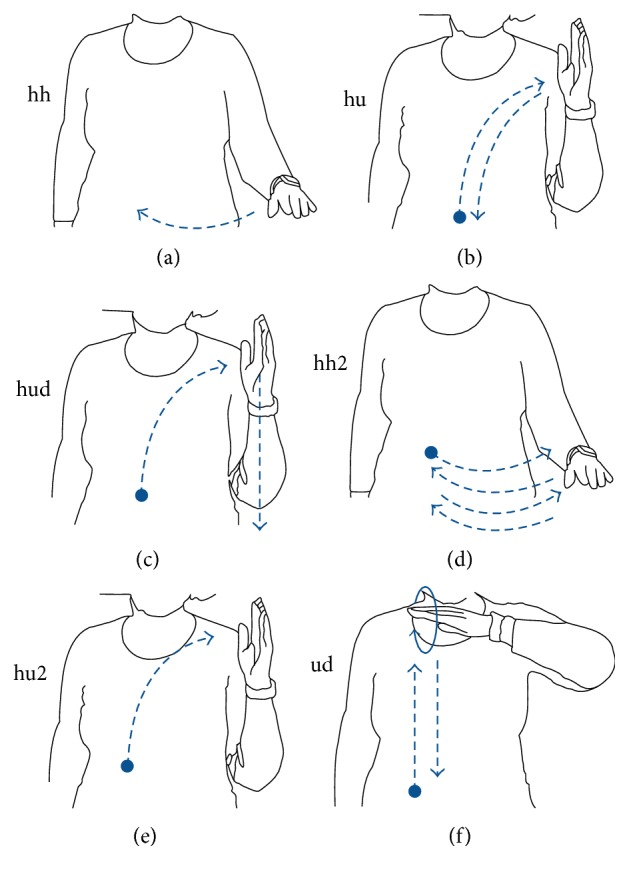
The selected gestures; a dot depicts a gesture start.

The gestures used for the evaluation of the system were selected according to the following criteria: they are characterized by the wrist movement, they are simple and natural gestures and they are easily repeated, they are different from each other, the gravity does not complicate their recognition and if possible contributes to their differentiation, and they can be related to commands for the manipulation of devices. The selected set is shown in Figure 1. The gesture “hh2” is twice the gesture “hh”, and the gesture “hu2” is twice the gesture “hu.” Nevertheless, the proposed system is flexible, and it can be trained to any kind of gesture dataset.

**Figure 2:**
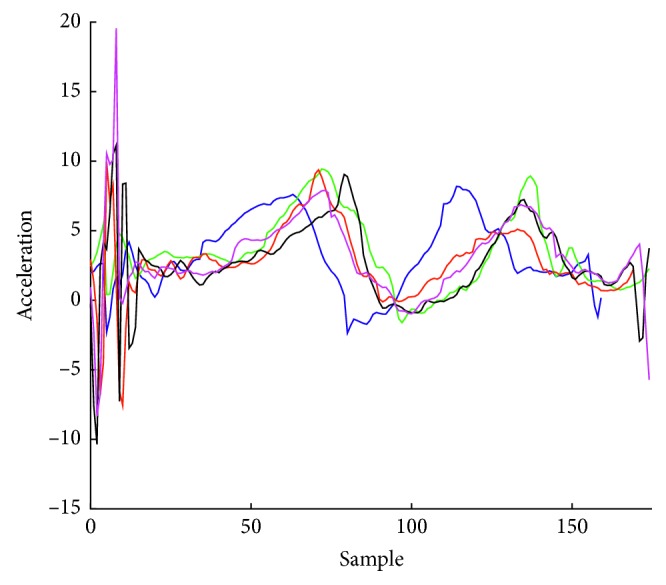
Raw measurements of a gesture performed by the same user 5 times.

The three axes along which acceleration is measured are bound to the watch. The accelerometer data received during a gesture include gravity and are also affected by the change of the orientation of the smartwatch during the movement. Data also contain noise. The use of tap event at the beginning and the end of the gesture affects the accelerometer data measurements at those points. Since the duration of a gesture varies and the accelerometer collects data at a constant rate, the number of samples for each gesture differs.  Figure 2 illustrates the raw accelerometer measurements along the *x*-axis of the smartwatch. The five curves in the figure correspond to 5 repetitions of the same gesture performed by one user. A heuristic algorithm to eliminate the effect of the tap event was used. Starting from the thirtieth measurement from the start and till the thirtieth measurement to the end, the maximum difference between two successive measurements was determined, in order to be used as a threshold. Starting from the thirtieth measurement from the start towards the first measurement, the difference between two successive measurements is calculated and if it exceeds the threshold value, the part of the gesture before that point is excluded. A similar procedure is applied to exclude a part of the time series near the end of it. In our work, we examined three alternative techniques for gesture recognition, which are discussed in the following subsections.

### 3.2. The Fast Fourier Transformation (FFT) Method

**Figure 3:**
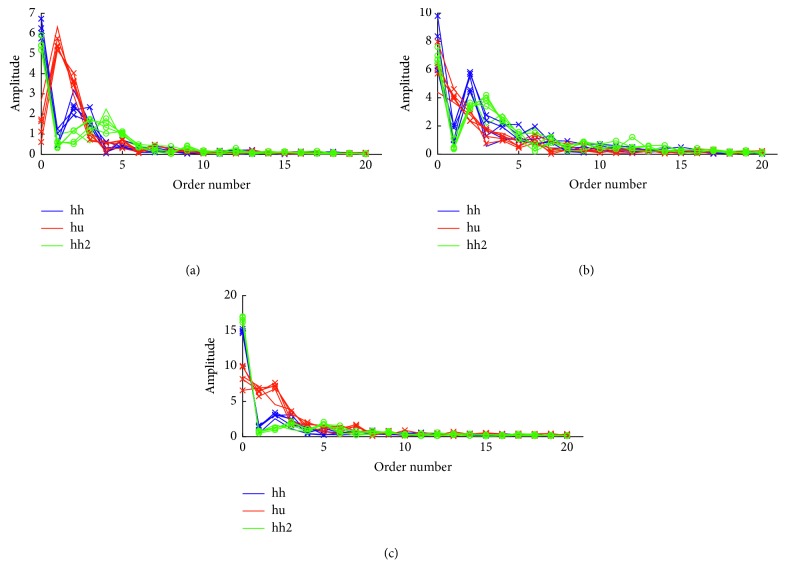
The FFT method: the final time series of 3 gestures performed by the same user 5 times each. (a) *x*-axis, (b) *y*-axis, and (c) *z*-axis.

**Figure 4:**
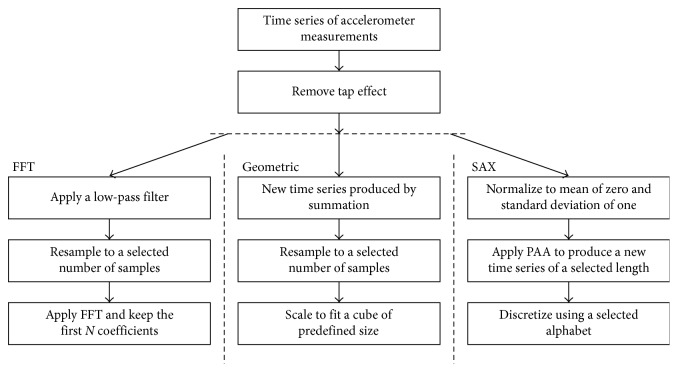
The preprocessing steps of the 3 alternative proposed methods.

FFT coefficients of motion signals can be used for gesture recognition according to the literature [32]. In the proposed method, a simple low-pass filter is initially applied as in [32]. The filter is recursively defined as following:

(1) $$\begin{array}{c} s_{t}=a\cdot x_{t-1}+\left(1-a\right)\cdot s_{t-1}, \end{array}$$

where *x*_*t*_ is the *t* order point of the time series and *s*_*t*_ the computed value at the *t* order point. For the constant *α* the value 0.3 was selected with trial and error during evaluation. In order that all-time series have the same length, data were resampled to 512 points using linear interpolation. We set the number of points to 512 so that it is a power of 2, as needed by the employed FFT implementation, and so that the original time series were not subsampled (given that these, in our datasets, had up to approximately 450 points). FFT was applied to the new time series, and the 21 first coefficients for each axis were kept. The latter value was chosen according to the findings of [32] and our preliminary experiments.  Figure 3 illustrates the final time series of 3 gestures performed by the same user 5 times each. The distance between two final time series was computed as the mean Euclidean distance of their points.  Figure 4 illustrates the preprocessing procedures implemented by the FFT method and the other two employed methods.

### 3.3. The Geometric Method

**Figure 5:**
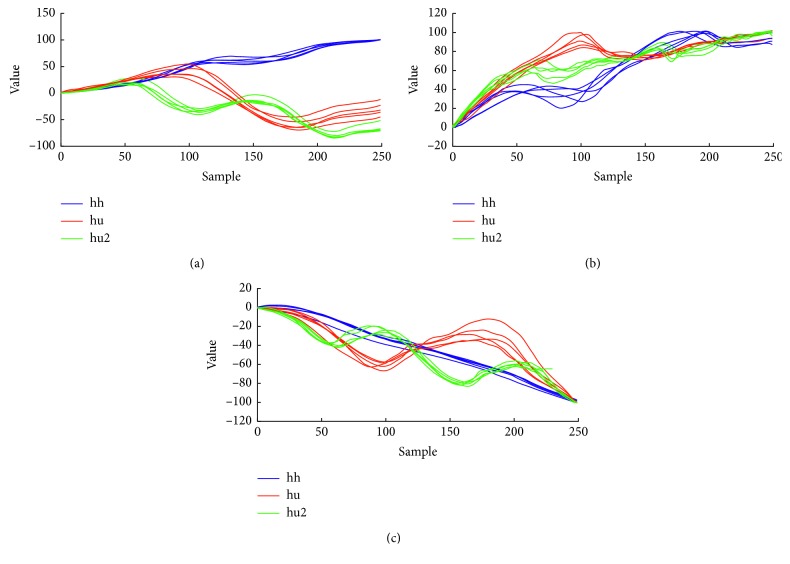
The geometric method: the final time series of 3 gestures performed by the same user 5 times each.

In the geometric method, captured motion and data were transformed to new time series by replacing a sample by the sum of its value and all the previous values. As previously, in order to maintain for all the time series the same length, data were resampled using linear interpolation. As a next step, the series was scaled to fit in a normalized cube of edge of 100 units as in [26], which means that after the scaling the maximum value minus the minimum value of samples at each axis was equal to 100.  Figure 5 illustrates the final time series of 3 gestures performed by the same user 5 times each. The distance between two final time series was computed as the mean Euclidean distance of their points.

### 3.4. The SAX Method

SAX (Symbolic Aggregate approXimation) [33–35] transforms a time series of arbitrary length *n* to a string of arbitrary length *w* using an alphabet of arbitrary size a. The time series is initially normalized to have a mean of zero and a standard deviation of one. Piecewise aggregate approximation is used to reduce the dimensionality from *n* to *w*. The “breakpoints” to be used for the discretization of the transformed time series are selected so that they divide the Gaussian distribution to equal probability areas. The time series is discretized to the selected alphabet by dividing the value space according to the determined “breakpoints” and replacing all the values that belong to a division with the same symbol of the alphabet. The distance between two symbols is defined to be zero if they differ at most one step. If they differ more steps, the distance is computed as the difference between the lower limit of the largest symbol minus the upper limit of the smallest symbol.

**Figure 6:**
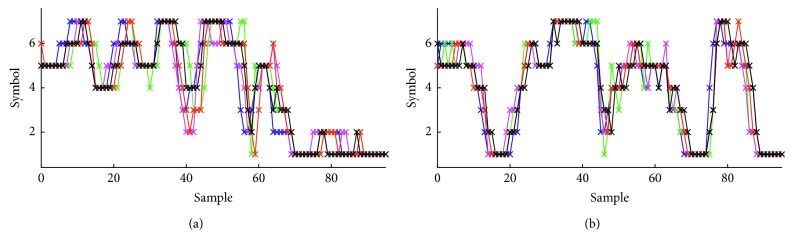
The SAX method: the final strings of 2 gestures, the hh gesture (a) and the hu gesture (b), performed by the same user 5 times each.

In order to apply the SAX method, we initially combined the data of the three axes in one time series, first all the *x*-axis data followed by all the *y*-axis data and the *z*-axis data. We then normalized each time series to have a mean of zero and a standard deviation of one, thus jointly normalizing the *x*, *y*, and *z*-axis data. The parameters of the SAX method we used were *w*=96 and *a*=7, which means we selected to represent a gesture with a string of 96 symbols (32 for each axis) using an alphabet of 7 symbols. The parameters were chosen so as to preserve the characteristics of the time series of the selected gestures. We transformed the normalized time series into the piecewise aggregate approximation (PAA) representation and after that we symbolized the PAA representation into a discrete string.  Figure 6(a) illustrates one gesture performed by the same user 5 times, and Figure 6(b) illustrates another gesture. The distance between two strings was computed as the mean distance of their symbols according to the SAX method.

The complexity of the preprocessing algorithm is O(*n*), where *n* is the length of the time series of the gesture measurements. The complexity of the recognition algorithm is O(*n*), where *n* is the parameter *w* of the SAX method.

## 4. The Proposed Gesture Recognition System

**Figure 7:**
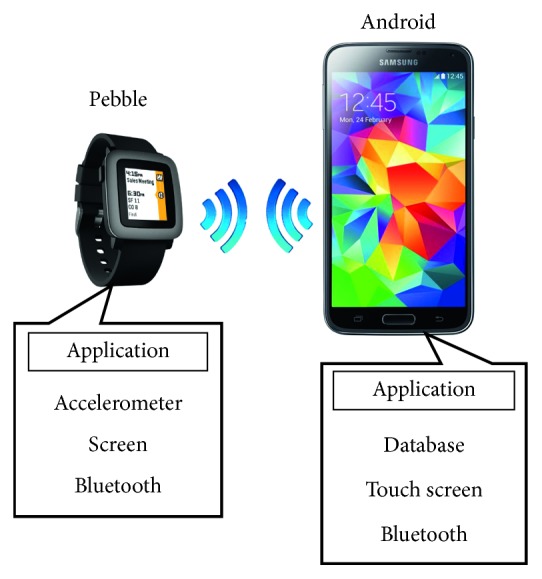
System architecture.

**Figure 8:**
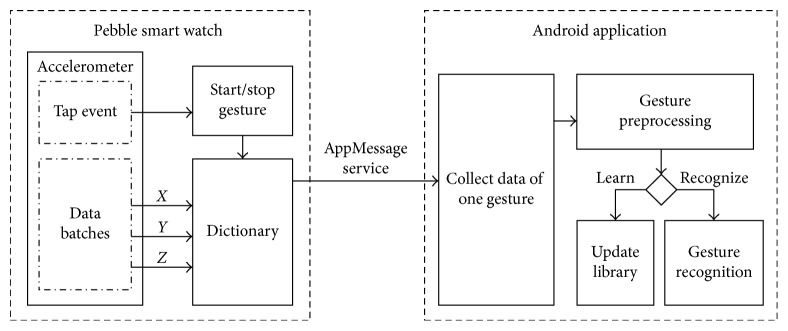
The communication between the Pebble and the Android modules.

The implemented system consists of two companion applications. The first application runs on the smartwatch (Pebble) and is responsible for capturing the accelerometer measurements and sending them to the Android application. The second application runs on the companion Android device. Android application provides the interface to the user, receives the motion data from the smartwatch, updates the database (template library) with the training gestures, and runs the recognition methods. Pebble and the Android device communicate with each other using Bluetooth.  Figure 7 illustrates the basic system architecture and the resources used.  Figure 8 illustrates the communication between the Pebble and the Android modules.

### 4.1. The Pebble Application

A significant design choice for the smartwatch application is the initiation. In our system, the tap event was selected to delimit the gesture so that the user can start and stop the gesture in a spontaneous and natural way. An abrupt movement of the wrist is perceived as tap event by Pebble so the user can start and stop the gesture using only the hand the smartwatch is worn on. Another important aspect is energy consumption. In order to save energy, the Pebble application is not constantly in operation but it is activated by the Android application. For the same reason accelerometer data are sent to Pebble application in batches. As soon as the Pebble application is activated, a message is displayed on its screen to inform the user for the Android application operation mode (training or recognizing). The Pebble application is waiting for a tap event after which it repeatedly sends accelerometer data to the Android application and receives the corresponding acknowledgment. A new tap event signifies the gesture end.

### 4.2. The Android Application

**Figure 9:**
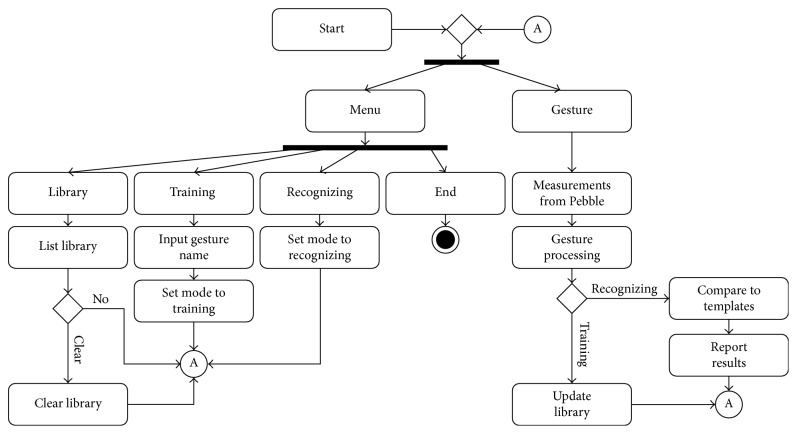
The Android application structure.

The Android application features a menu with commands where the user can manage the gesture library. The user can see how many templates are stored in the library and manage them (i.e., delete a template or clear the library). Additional commands refer to training (i.e., set the application in training mode) and recognizing (i.e., set the application in recognizing mode and let the user to key in the gesture name). The user can start the Pebble application, using a button, in order to receive accelerometer measurements. In recognizing mode, after the end of the gesture, the application answers with the best match obtained by each recognition method.  Figure 9 illustrates the Android application structure.

## 5. The System in Practice—Use Case and Experimental Results

### 5.1. Use Case

Gestures may be considered as a means of multimodal interaction. An older person may have health problems pertaining to his or her fingers. Using the small buttons of a remote controller is an issue and an alternative way to control the television, the air conditioner, the window blinds, and so on is required. A set of gestures that takes into consideration the mobility decline is the perfect solution. As an example, the older person can use “hu” gesture to change the tv program to the next channel and “hh” to change it to the previous channel. Additional commands may be correlated for controlling with gestures wheelchairs [36] and robotic platforms intended for assisted living.

### 5.2. Evaluation

In order to evaluate the application and the utilized methods, we asked four persons to perform the gestures illustrated in Figure 1. Each set included at least 5 repetitions of the same gesture. A few days later, the same persons performed additional gesture sets. Finally, we collected 8 sets with a total of 304 gestures. Two types of evaluation were performed; one involving training by the same person and a second utilizing training by different users, in order to assess the robustness of the proposed methods.

#### 5.2.1. User-Dependent Recognition (Personalized Gesture Recognition)

In this case, the first gesture set of user A was used to train the system and the second set of the same user for testing. This process was repeated using the second set to train the system and the first for testing. The abovementioned process was repeated for all users, and the average values are reported in Table 2 for the three recognition methods. More specifically, the geometric method produced correct results in the 93% of the experiments with the success rate ranging from 83% to 100%. The FFT method produced correct answer in the 95% of the tests with the success rate ranging from 88% to 100%, while the SAX method outperforms producing correct response in the 99% of the occasions with the success rate ranging from 94% to 100%. Tables 3–5 present the confusion matrices of the methods. On the first row, there are the names of the gestures the user performed and on the first column the system response.

#### 5.2.2. User Independent Recognition

In order to evaluate the system in the case of the user independent recognition the following process was applied: the first set of user A was used to train the system. All the gestures of the other users (B, C, and D) were used as the gestures to be recognized. The same process was repeated three times using the first set for the other users (B, C, and D) as the training set. The results are summarized in Table 6. The geometric method produced correct results in the 84% of the occasions in average with the lowest success rate to be 69%. The FFT method produced correct answer in the 71% of the occasions in average with the lowest success rate to be 42%. The SAX method produced correct answer in the 95% of the experiments in average with the lowest success rate to be 86%. SAX outperforms in the user independent recognition case as well achieving quite satisfactory accuracy. Thus, it can be assumed that the SAX method can be utilized in user independent recognition in real applications.

### 5.3. Battery Consumption

The gesture duration or equivalently the time series length varies considerably depending on gesture type and user. This has the effect that Pebble battery consumption caused by the collection and transmission of the motion data will be different depending on gesture type and user. Pebble reports battery drop in steps of 10%. In order to estimate how the use of the gesture recognition application affects the Pebble autonomy we asked two of the users to perform repeatedly the gestures of Figure 1 until the battery indicator dropped by 10%. A drop from 100% to 80% battery resulted after 780 gestures. Taking into consideration that the Pebble battery duration for normal use is 7 days, the expected battery life *D*, measured in days, is given by the expression (2). In this expression, *w* stands for the average fraction of the battery consumed per gesture and *N* stands for the number of gestures performed per day.

(2) $$\begin{array}{c} D=\frac{1}{\left(1/7\right)+w\cdot N}. \end{array}$$

**Figure 10:**
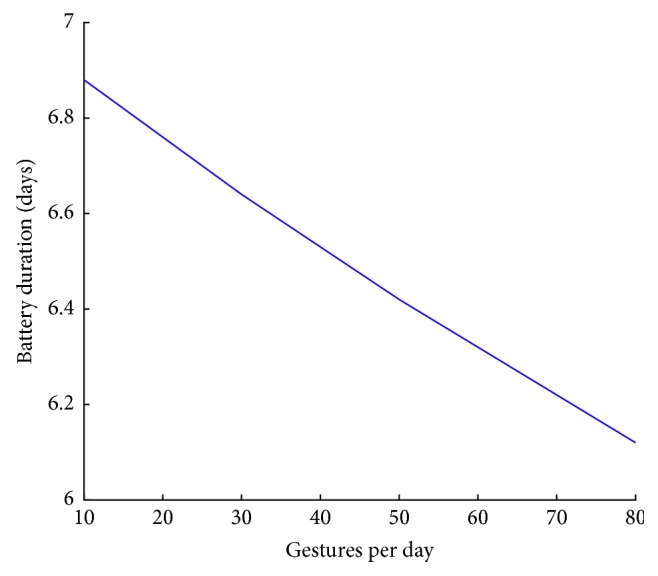
Estimated battery life. Horizontal axis is the number of detected gestures per day. Vertical axis shows the effect on battery life.

The estimated battery duration is illustrated in Figure 10. The autonomy remains above 6 days even under the high use of 80 gestures per day.

### 5.4. Usability Assessment

The challenges that elderly users experience with user interfaces are related to physical issues, technology experience, and cognitive issues [37]. For example, deteriorating physical condition results in impaired eyesight, haptic deterioration, or reduced hearing. Designing for the elderly, we have to take into consideration the specific challenges they face. Although the main focus of this work is on technical innovation and not on usability assessment, we evaluated the implemented system from this perspective as well. There are many assessment techniques to evaluate Ambient Assisted Living (AAL) technologies. Salvi et al. [38] propose a methodological framework for evaluating AAL solutions. They present both subjective and objective evaluation techniques. Colomer et al. [39] present their experience in evaluating AAL solutions in the Smart House Living Lab. They describe the evaluation methodologies used in their projects and propose a set of guidelines to conduct evaluations. Queirós et al. [40] present a systematic literature review of AAL technologies, products, and services regarding usability and accessibility. Finally, Holzinger et al. [41] concluded in their research that the level of education and the previous exposure to technology are relevant to the acceptability of new technology by the elderly.

In this work, in order to investigate the acceptance and usability of the proposed system, we collected the opinion of 20 users, 10 males and 10 females, of age 63 to 72. We used questionnaires based on a modified System Usability Scale (SUS) [42]. SUS is a mature questionnaire, it is considered to be very robust and flexible, and it has been extensively used and adapted. In this context, we modified the SUS statements to clearly describe the system we evaluate.

More specifically, SUS is a Likert scale. It is composed of ten statements, five positive statements and five negative statements which alternate. The respondent indicates the degree of disagreement or agreement with the statement on a 5-point scale varying from 1, strongly disagree, to 5, strongly agree. For statements 1, 3, 5, 7, and 9 that are positively worded statements the score is calculated by the scale position minus 1. For statements 2, 4, 6, 8, and 10 that are negatively worded the score is 5 minus the scale position. Each statement's score ranges from 0 to 4. The sum of the scores of the ten statements is multiplied by 2.5 to produce the overall value of SUS on the scale 0 to 100. The level of education and the previous exposure to technology as proposed by Holzinger et al. [41] were not included in the questionnaire, since the majority of the participants share the same characteristics. However, we intend to include them in a future more thorough evaluation of the proposed application.

The results of the evaluation are depicted in Table 7, which presents the statements used and the mean values of the collected answers and scores. The total result of 78/100 is quite encouraging and it demonstrates the potential of the proposed system. In the participants' responses, no gender-related differences were observed. These two parameters must also be investigated for the proposed system.

## 6. Conclusions

In this work, we examined the feasibility of a gestured recognition system based on a smartwatch and a connected smartphone. The motion measurements obtained by the accelerometer of the smartwatch during a gesture were captured and relayed to the smartphone. Three alternative recognition methods were implemented. The SAX method outperformed the rest and produced high accurate results even in the case of user independent training.

Although the gestures used to evaluate our methods were chosen due to their simplicity, the application we developed can be utilized with different gesture sets. The critical condition for a gesture set to be suitable is that the gestures must be quite different from each other in accordance with the combination of the orientation of the watch and the direction of its movement. Gestures following similar trace with different orientation of the watch are considered to be different gestures. In addition the accelerometer measurements contain gravity, and a suitable set of gestures must exploit the contribution of the gravity to the diversification of the gestures. The reported results are quite encouraging and prove the efficiency of a plain smartwatch to detect simple gestures.

There are several interesting issues related to the improvement of the result reliability to achieve the goal of absolutely reliable recognition. If the gesture set is known in advance, the recognition method can be adapted to make use of those characteristics of the gestures that differentiate from each other.

An extension of the training process could be the choice of the most successful gesture recognition method between those implemented by the application. During an initial training phase, more than one recognition method will be used, and the user will confirm or reject the application answers. Depending on the correct results, the most successful method will be selected by the application for future use.

The way a user makes a gesture may change over the time. An open issue is whether a dynamic update of the library could be applied.

The development of a universal vocabulary, which is an intermediate level between the gesture recognition application and the device control, is also of interest. Some commands like increasing, reducing, continuing, stop, redo, and so on could compose a standard vocabulary for handling everyday devices. A user could have his own set of gestures to implement the commands that are included in the vocabulary.

In a future work, we intend to incorporate the abovementioned ideas to create an application to facilitate people with disabilities in daily life activities.

## Figures and Tables

**Table 1:** Summary of gesture recognizers.

Gesture recognizer	Data dimensions	Scaling	Metric
$1	2D	Yes	Euclidean
Protractor	2D	No	Cosine
$3	3D	Yes	Euclidean
Protractor3D	3D	Yes	Euclidean
$N	2D	Yes	Euclidean
$N-protractor	2D	Yes	Cosine
$P	2D	Yes	Other
uWave	3D	No	DTW

**Table 2:** Results of personalized gesture recognition.

Library-recognized	Geometric			FFT			SAX		
	Correct	False	%	Correct	False	%	Correct	False	%
A1-A2	30	6	83	32	4	89	35	1	97
A2-A1	29	1	97	29	1	97	30	0	100
B1-B2	47	3	94	44	6	88	50	0	100
B2-B1	29	1	97	29	1	97	30	0	100
C1-C2	33	3	92	36	0	100	34	2	94
C2-C1	29	6	83	34	1	97	35	0	100
D1-D2	50	0	100	49	1	98	50	0	100
D2-D1	37	0	100	36	1	97	37	0	100
Totals	284	20	93	289	15	95	301	3	99

**Table 3:** Geometric method: confusion matrix.

Geometric	hh	hu	hud	ud	hh2	hu2
hh	49			1		
hu		43	4			3
hud		1	46	5		1
ud			2	42		
hh2					52	1
hu2		1	1			46

**Table 4:** FFT method: confusion matrix.

FFT	hh	hu	hud	ud	hh2	hu2
hh	47			1	6	
hu		43	1			
hud		2	52	3		
ud				44		
hh2	2				44	
hu2						51

**Table 5:** SAX method: confusion matrix.

SAX	hh	hu	hud	ud	hh2	hu2
hh	49					
hu		43				
hud		2	52			
ud			1	48		
hh2					52	
hu2						51

**Table 6:** User independent recognition.

Method	Correct	False	%	Min %
Geometric	636	123	84	69
FFT	542	217	71	42
SAX	732	27	96	86

**Table 7:** Questionnaires' results.

	Modified SUS statements	Answer	Score
1.	I think that I would like to use a smartwatch to control devices frequently	3,7	2,7
2.	I found the system of the smartwatch and the smartphone to control devices unnecessarily complex	1,6	3,4
3.	I thought that performing gestures to control devices was easy	3,8	2,8
4.	I think that I would need the support of a technical person to be able to use this system	1,3	3,7
5.	I found the various functions in this system (i.e., the training with user-selected gestures and the recognition of gestures) were well integrated	4,1	3,1
6.	I thought there was too much inconsistency in this system and I expect it to fail	1,8	3,2
7.	I would imagine that most people would learn to use such a system very quickly	4,1	3,1
8.	I found that the system of performing gestures to control devices was very cumbersome to use	1,7	3,3
9.	I feel very confident that I will be able to use the system	3,9	2,9
10.	I think I need to learn a lot of things before I can use this system	2,0	3,0
	Total score	—	31,2
	SUS score	—	78,0

## Data Availability

The dataset used in this research is available to the research community upon e-mail request to the corresponding author.
